# Strategies for Reducing Antimicrobial Use in Cattle Through Gut Microbiome Modulation: A Systematic Review of Alternatives to Antibiotics

**DOI:** 10.3390/ani16121850

**Published:** 2026-06-15

**Authors:** Zanoxolo Ntsongota, Olusegun Oyebade Ikusika, Mthunzi Mndela, Ishmeal Festus Jaja

**Affiliations:** 1 Department of Animal and Pasture Sciences, Faculty of Science and Agriculture, University of Fort Hare, Alice 5700, South Africa; 2 SAMRC Microbial Water Quality Monitoring Centre, University of Fort Hare, Alice 5700, South Africa; 3 Department of Agriculture and Animal Health, University of South Africa, Johannesburg 1709, South Africa

**Keywords:** cattle, antimicrobial, gut microbiome, natural feed additives

## Abstract

The escalating global threat of antimicrobial resistance (AMR) has intensified efforts to identify safe, effective, and sustainable alternatives to in-feed antibiotics in livestock production. The bovine gastrointestinal microbiome plays a central role in host immunity, nutrient utilization, and disease resilience, positioning microbiome-modulating interventions as promising candidates for antimicrobial stewardship. Despite growing experimental interest, a systematic synthesis of the available evidence in cattle is lacking. This systematic review aimed to evaluate the efficacy of microbiome-modulating interventions, including probiotics, prebiotics, postbiotics, phytogenic feed additives, essential oils, organic acids, and native rumen microbial supplements, as strategies to reduce antimicrobial use in cattle, and to characterize their effects on gut microbial diversity, fermentation characteristics, and host health and performance outcomes. A systematic search of Scopus, Web of Science, and EBSCOhost (including Academic Search Ultimate, MEDLINE with full text, and CAB Abstracts with Full text) was conducted in accordance with PRISMA guidelines. Studies were eligible if they used cattle (dairy cattle, beef cattle, calves, or mixed production systems), employed a microbiome-modulating intervention, and reported at least one microbiological or host outcome. Seventeen peer-reviewed studies published between 2010 and 2025 were included after full-text screening. Risk of bias was assessed using an adapted SYRCLE tool, which identified moderate overall study quality; the majority of included studies were randomized controlled trials or controlled experiments, though reporting of allocation concealment and blinding was inconsistent across studies. Across the 17 included studies, five broad categories of interventions were evaluated: probiotics (n = 5 studies), prebiotics (n = 2), postbiotics and organic acids (n = 4), phytogenic additives and essential oils (n = 4), and native rumen microbial supplements (n = 2). Animals spanned neonatal dairy calves, weaned Holstein calves, dairy heifers, lactating dairy cows, and *Bos indicus* feedlot beef cattle. Probiotics and organic acids most consistently improved growth performance: benzoic acid supplementation increased average daily gain by 8.4% (*p* < 0.05) and fructo-oligosaccharide prebiotics elevated body weight at weaning by 6.7% (*p* < 0.01). Native rumen microbial supplements improved energy-corrected milk yield by up to 3.1% without increasing dry matter intake. Polyphenols and bile acids demonstrated the strongest immunological and disease-preventive effects, reducing calf mortality by approximately 40% and disease severity by approximately 35%, respectively. Microbiome analyses revealed intervention-dependent increases in microbial diversity and shifts toward taxa associated with improved fermentation efficiency, including enrichment of propionate-producing *Prevotellaceae*, butyrate-associated *Ruminococcus*, and hindgut *Bifidobacterium*. Rumen fermentation outcomes included reductions in the acetate:propionate ratio and ammonia-N concentrations and improvements in fiber digestibility of 3.6–4.4 percentage units in dairy cows. Phytogenic additives preserved microbial diversity without inducing broad-spectrum suppression, functioning primarily as microbiome stabilizers rather than direct antimicrobial replacements. This systematic review provides evidence that gut microbiome modulation may enhance growth performance, improve fermentation efficiency, and reduce disease susceptibility in cattle, thereby supporting antimicrobial use reduction across dairy, beef, and mixed production systems. Effect magnitudes varied substantially across intervention categories and production contexts, and study quality was moderate, underscoring the need for larger, pre-registered trials with standardized outcome reporting and direct antibiotic comparator arms. Probiotics, prebiotics, and bile acid metabolites showed the greatest potential as components of integrated antimicrobial stewardship strategies in cattle production.

## Simple Summary

The rising concern over antimicrobial resistance has intensified the search for effective strategies to reduce antibiotic use in cattle production. Gut microbiome modulation has emerged as a promising approach to enhance animal health, productivity, and disease resilience without relying on antibiotics. This systematic review critically evaluates 17 high-quality studies published over the past 15 years to assess the potential of natural gut-health-promoting supplements, including probiotics and plant-based additives, as alternatives to antibiotics in cattle production systems. The findings demonstrated that these supplements can improve growth performance, feed digestion, and disease resistance, thereby significantly reducing the reliance on antibiotics. However, the effectiveness of these interventions varies depending on factors such as cattle type, production system, and supplementation dosage, highlighting the importance of tailored application strategies for optimal outcomes.

## 1. Introduction

Antimicrobials have long been integral to cattle production systems for the prevention and treatment of infectious diseases and, historically, for growth promotion [1]. However, the extensive use of antibiotics in livestock has been strongly associated with the development and dissemination of antimicrobial resistance (AMR), which poses a major threat to animal health, food safety, and public health worldwide [2,3,4]. Resistant bacteria and antimicrobial resistance genes originating from cattle production systems may persist in the environment and be transmitted through direct contact or via the food chain, underscoring the need for improved antimicrobial stewardship in animal agriculture [5]. In response to rising AMR concerns, regulatory frameworks in many regions have increasingly restricted antimicrobial use in food-producing animals, including the prohibition of antibiotic growth promoters and tighter controls on prophylactic and metaphylactic applications [6,7,8]. While these measures are essential for safeguarding public health, they present challenges for cattle producers who must maintain animal health, welfare, and productivity under reduced antimicrobial availability. Consequently, there is growing interest in developing effective and sustainable alternatives to antibiotics that support disease resilience and production efficiency in livestock production [9,10,11,12]. The gut microbiome plays a pivotal role in cattle health and productivity, influencing feed digestion, nutrient utilization, immune function, and resistance to enteric pathogens [12,13]. In ruminants, the rumen microbiome is central to fermentation and energy supply, while the hindgut microbiota contributes to immune modulation and gut barrier integrity [14,15]. Disruptions to these microbial communities can compromise gut health and increase disease susceptibility, often leading to increased antimicrobial interventions [16]. Conversely, targeted modulation of the gut microbiome has emerged as a promising strategy to enhance microbial stability, promote beneficial microorganisms, and suppress pathogenic populations.

A range of microbiome-based interventions have been investigated as alternatives to antimicrobial use in cattle, including probiotics, prebiotics, synbiotics, postbiotics, phytogenic feed additives, organic acids, enzymes, and microbial-derived products. These strategies aim to improve gut microbial composition and functionality, enhance host immune responses, and improve growth performance and feed efficiency. Importantly, by reducing pathogen load and disease incidence, such interventions may lower the need for antimicrobial treatments and reduce selection pressure for AMR within cattle production systems [1]. Despite the expanding body of research on gut microbiome modulation in cattle, results remain inconsistent across studies due to differences in animal age, production system, dietary context, intervention type, and study design. Furthermore, the extent to which microbiome-based strategies can consistently reduce antimicrobial use while maintaining or enhancing animal performance is not fully understood. A systematic synthesis of the available evidence is therefore required to clarify the effectiveness of these approaches and identify knowledge gaps.

The objective of this systematic review is to evaluate strategies aimed at reducing antimicrobial use in cattle through gut microbiome modulation. Specifically, this article presents a systematic review focused on microbiome-based strategies as alternatives to antibiotics in cattle production systems, with the broader goal of reducing antimicrobial use and mitigating antimicrobial resistance. The review explores how modulation of the bovine gut microbiome through probiotics, prebiotics, postbiotics, phytogenic feed additives, essential oils, organic acids, and native microbial supplements may improve animal health, productivity, gut stability, and disease resilience without relying on conventional antimicrobials.

## 2. Methods and Materials

The systematic literature search was conducted in accordance with the PRISMA guidelines (Preferred Reporting Items for Systematic Reviews and Meta-Analyses) [17]. The searches were performed in multiple academic databases, including Scopus, Web of Science, and EBSCOhost (Academic Search Ultimate, MEDLINE with full text, and CAB Abstracts with Full text), covering the period from 1 January 2010 to 31 December 2025, as summarized in Table 1. Search strategies were independently adapted for each database to conform to platform-specific indexing vocabulary, controlled terminology, and field-tag syntax. Web of Science employed Topic (TS=) field codes with truncation; Scopus used TITLE-ABS-KEY field codes; EBSCOhost (Academic Search Ultimate, MEDLINE with full text, and CAB Abstracts with Full text) searches utilized MeSH controlled vocabulary combined with free-text [tiab] terms. Only peer-reviewed articles published in English were considered. Non-primary literature, including review articles, book chapters, short communications, conference proceedings, letters, and editorials, was excluded to ensure methodological rigor and focus on original research. The restriction on English-language studies was due to their dominance in scientific publishing, indexing consistency, and practical constraints such as translation resources. All references were first exported to EndNote for deduplication and consolidation and then imported into Covidence software for title and abstract screening, followed by full-text evaluation. The search string used for each database is presented in Table 1 below.

**Inclusion Criteria**

- **Population**
  ○ Studies conducted in cattle (beef and/or dairy);
  ○ Includes calves, heifers, steers, and adult cattle;
  ○ Healthy animals or cattle under disease or production stress.
- **Intervention**
  ○ Strategies aimed at modulating the gut microbiome to reduce antimicrobial use;
  ○ Includes:
    ▪ Probiotics;
    ▪ Prebiotics;
    ▪ Synbiotics;
    ▪ Phytogenics/plant extracts;
    ▪ Essential oils;
    ▪ Organic acids;
    ▪ Enzymes;
    ▪ Yeast-based products;
    ▪ Postbiotics or microbial metabolites;
    ▪ Dietary or feed-based microbiome interventions.
- **Comparator**
  ○ Antibiotic or antimicrobial treatments;
  ○ Negative control (no additive);
  ○ Placebo or standard diet control.
- **Outcomes**
  ○ Reduction or replacement of antimicrobial use;
  ○ Changes in gut microbiota composition, diversity, or function;
  ○ Animal health outcomes (disease incidence, immune response, diarrhea);
  ○ Growth and performance parameters (average daily gain, feed conversion ratio);
  ○ Indicators of antimicrobial resistance (where reported).
- **Study Design**
  ○ Randomized controlled trials;
  ○ Controlled feeding trials;
  ○ Field trials;
  ○ Cohort or longitudinal studies.
- **Publication Characteristics**
  ○ Peer-reviewed journal articles;
  ○ Published in English;
  ○ Published within the defined review period (2010–2025).

**Exclusion Criteria**

- **Population**
  ○ Studies involving non-cattle species (poultry, pigs, sheep, goats, humans);
  ○ In vitro or laboratory-only studies without in vivo cattle validation.
- **Intervention**
  ○ Studies evaluating antibiotics only, with no alternative strategy;
  ○ Vaccination-only strategies not linked to gut microbiome modulation;
  ○ Chemical or pharmaceutical interventions unrelated to microbiota.
- **Outcomes**
  ○ No relevant outcomes related to antimicrobial reduction or gut microbiome
  ○ Studies reporting only product quality (milk yield/composition) without health or microbiome outcomes
- **Study Design**
  ○ Review articles or meta-analyses;
  ○ Conference abstracts, editorials, commentaries;
  ○ Theses, dissertations, or book chapters;
  ○ Case reports without a comparator.
- **Publication Characteristics**
  ○ Non-peer-reviewed sources;
  ○ Articles not available in full text;
  ○ Publications in languages other than English;
  ○ Duplicate studies.

### Risk-of-Bias Assessment

The risk of bias of the included animal studies was assessed using the SYRCLE (Systematic Review Centre for Laboratory Animal Experimentation) risk-of-bias tool, which is specifically adapted from the Cochrane framework for experimental animal intervention studies. The appraisal encompassed ten domains of potential bias, including selection bias (sequence generation, baseline characteristics, and allocation concealment), performance bias (random housing and blinding of caregivers and investigators), detection bias (random outcome assessment and blinding of outcome assessors), attrition bias (incomplete outcome data), reporting bias (selective outcome reporting), and other sources of bias. Each domain was judged as presenting a low, high, or unclear risk of bias based on the information provided in the published reports. In cases where methodological details were insufficiently described, the risk of bias was classified as unclear, consistent with SYRCLE guidelines. An overall qualitative risk-of-bias profile was generated for each study to support the interpretation of microbiota, fermentation, and animal performance outcomes. To enhance transparency, disagreements between reviewers during the risk-of-bias assessment were resolved through discussion until consensus was reached.

## 3. Results

### 3.1. Characteristics of the Included Studies

**Figure 1:**
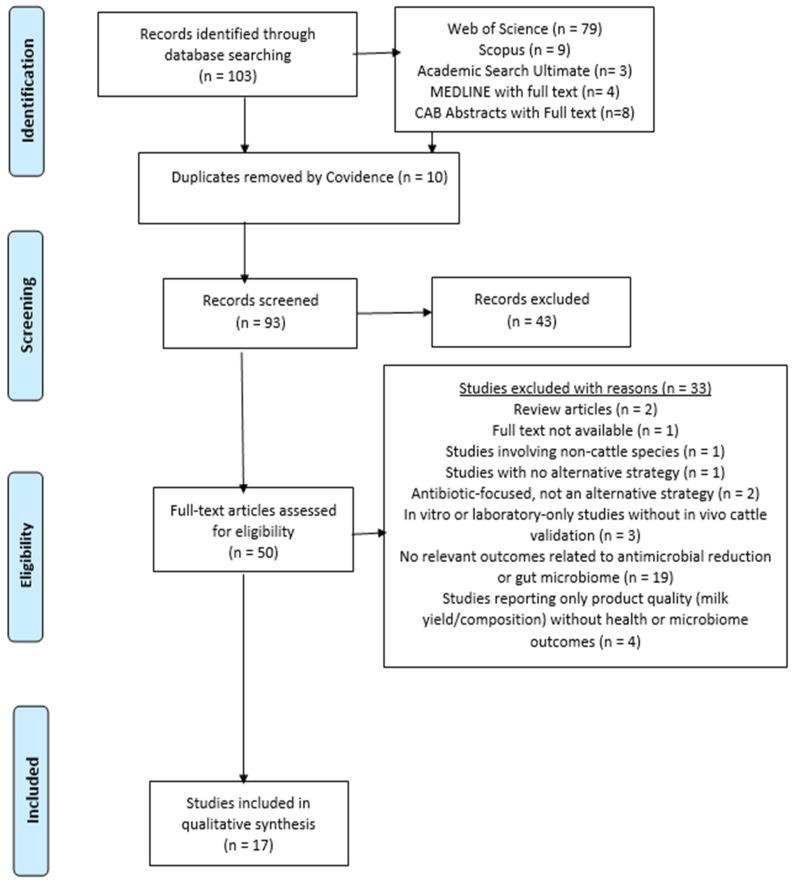
The PRISMA flow diagram illustrates the systematic process of study identification, screening, eligibility assessment, and inclusion for the current review.

The results of the systematic search process are illustrated in Figure 1. The initial search yielded a total of 103 records from the following databases: Scopus (n = 9), Web of Science (n = 79), and EBSCOhost (including Academic Search Ultimate (n = 3), MEDLINE with full text (n = 4), and CAB Abstracts with Full text (n = 8)). Following the retrieval, a total of 10 duplicate records were identified by Covidence and removed. The remaining 93 records were subjected to title and abstract screening, which resulted in the exclusion of 43 studies. Full-text screening was subsequently performed on the remaining 50 articles. During this stage, 33 articles were excluded according to the established eligibility criteria. Consequently, 17 studies were selected for inclusion in the final review. In addition, manual reference screening and citation tracking of the included studies were conducted to identify potentially relevant articles that may not have been captured through the database searches.

### 3.2. Synthesis of Findings of Included Studies

Table 2 shows the summary of the seventeen studies included in the present systematic study. The included studies were only conducted in vivo feeding trials conducted under controlled experimental conditions. Study populations encompassed neonatal calves, pre-weaned and weaned dairy calves, growing heifers, feedlot cattle, lactating dairy cows, and dry cows, allowing for a comprehensive evaluation of microbiota modulation across physiological stages [18,19,20,21,22,23,24,25,26,27,28,29,30,31,32,33,34]. Most trials employed randomized or completely randomized designs, with intervention durations ranging from short-term neonatal studies (2–4 weeks) to longer lactation or feedlot trials (8–16 weeks) [20,21,22,24,27,34]. Microbial community profiling was commonly performed using 16S rRNA gene sequencing, although several studies integrated metagenomic, metabolomic, and fermentation analyses to elucidate functional microbial responses [18,19,20,21,22,23,24,25,26,27,28,29,30,31,32,33,34]. The gastrointestinal compartments evaluated included the rumen, hindgut, and fecal microbiota, with several studies assessing more than one compartment, reflecting growing recognition of the interconnected nature of the bovine gastrointestinal microbiome.

Dietary interventions are commonly classified into several functional groups, including probiotics and direct-fed microbials, prebiotics, postbiotics, phytogenic compounds and botanical extracts, essential oils, and organic acids. These feed additives act through different mechanisms to modulate the gut microbiota, enhance intestinal health, and improve growth performance in livestock.

**Figure 2:**
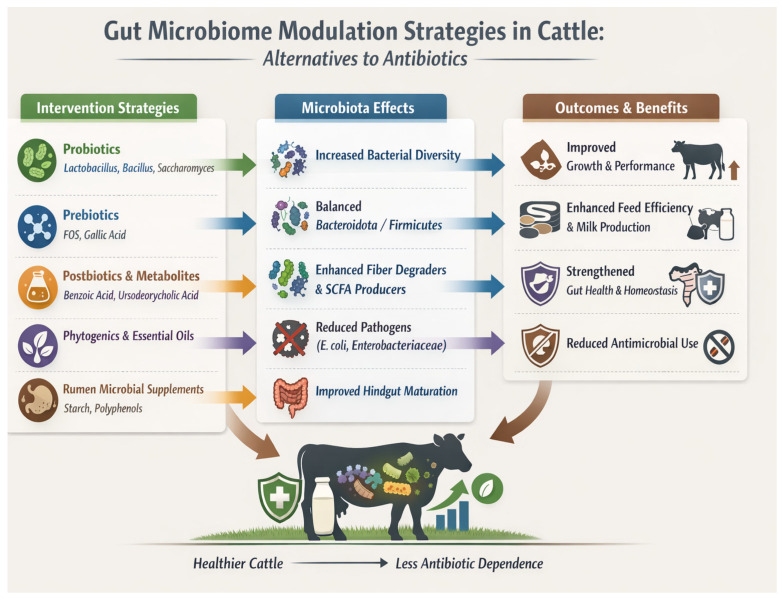
Overview of gut microbiome modulation strategies in cattle, highlighting intervention approaches, microbial responses, and associated outcomes aimed at improving gut health and productivity while reducing reliance on antimicrobial use.

Figure 2 shows pictures of various intervention strategies, microbiota effects, and outcomes and benefits in dairy cows. These interventions were tested either individually or in combination with standard feeding regimes such as milk replacers, starters, high-concentrate diets, or forage-based diets. Studies evaluating rumen microbiota consistently reported diet-induced shifts in bacterial community structure, particularly in response to phytogenic compounds, essential oils, and organic acids [18,19,22,23]. Supplementation with essential oils frequently increased the relative abundance of *Prevotellaceae* and other propionate-producing taxa, accompanied by improved rumen fermentation profiles, including increased propionate concentrations and more stable rumen pH [22]. Organic acid supplementation, including benzoic and dicarboxylic acids, influenced rumen microbial diversity and selectively enriched taxa associated with starch degradation and energy metabolism, especially in animals fed high-grain diets [19]. Metagenomic analyses demonstrated that such interventions promoted functional redundancy and metabolic adaptability, suggesting enhanced microbial resilience under nutritionally challenging conditions [21]. Prebiotic and probiotic interventions exerted pronounced effects on the hindgut and fecal microbiota, particularly in neonatal and pre-weaned calves [27,34]. Fructo-oligosaccharide supplementation consistently increased Bifidobacterium abundance and supported the maturation of hindgut microbial communities, indicating a beneficial role in early-life microbial colonization [33]. Probiotic lactic acid bacteria and postbiotic products derived from *Saccharomyces cerevisiae* fermentation stabilized fecal microbial communities, reducing inter-individual variability and mitigating dysbiosis during dietary transitions [21,27]. These effects were particularly evident during periods of physiological stress, such as weaning or exposure to high-starch diets.

Several studies specifically addressed microbial responses under stress or challenge conditions, including grain-induced subacute ruminal acidosis (SARA) and pathogenic *Escherichia coli* infection [23,34]. Postbiotic supplementation was shown to stabilize rumen liquid microbiota during SARA, maintaining microbial diversity and limiting the proliferation of acid-tolerant, potentially detrimental taxa [21]. Studies investigating gallic acid and gut microbiota-derived ursodeoxycholic acid in neonatal calves demonstrated protective effects against extended-spectrum β-lactamase-producing enteroaggregative *E. coli* [20,34]. These interventions modulated gut microbial composition, enhanced intestinal barrier integrity, and reduced inflammatory markers, highlighting the functional role of microbial metabolites in disease resistance [20,34]. Early-life interventions emerged as a critical theme across studies. Modulation of rumen microbiota during the neonatal period influenced subsequent fecal microbiota colonization dynamics, suggesting long-term effects of early dietary strategies [21]. A study assessing early-life rumen modulation reported carry-over effects on gut health, with animals exhibiting more stable microbial communities and improved gastrointestinal function later in life [26]. These findings support the concept of microbial programming, whereby targeted nutritional interventions during early development shape the trajectory of gastrointestinal microbiota establishment and functional capacity [35].

Across life stages, changes in microbial composition and diversity were frequently associated with improvements in animal performance metrics. Probiotic and direct-fed microbial supplementation enhanced energy-corrected milk yield and feed efficiency in lactating dairy cows, while prebiotic and phytogenic interventions improved growth performance in calves [25,28,30]. A feedlot study demonstrated that microbiota modulation was linked to enhanced metabolic efficiency and carcass responses, particularly when probiotics were included in high-concentrate diets [24]. Although causal relationships cannot be definitively established, the consistency of associations across studies strengthens the biological plausibility of microbiota-mediated performance benefits. While several studies focused exclusively on either rumen or hindgut microbiota, an increasing number of investigations highlighted cross-compartment interactions. Dietary interventions influencing rumen fermentation often resulted in downstream effects on hindgut microbial composition, emphasizing the need for an integrated view of the bovine gastrointestinal microbiome [23]. Collectively, the evidence demonstrates that non-antibiotic dietary interventions consistently modulate bovine gastrointestinal microbiota in a direction associated with improved fermentation efficiency, microbial stability, and animal health outcomes. However, heterogeneity in study design, intervention formulations, and microbiome analytical approaches limits direct quantitative comparisons. Notably, few studies employed long-term follow-up or functional multi-omics approaches, highlighting a critical gap in understanding the durability and mechanistic basis of microbiota modulation.

### 3.3. Assessment of Risk of Bias

**Figure 3:**
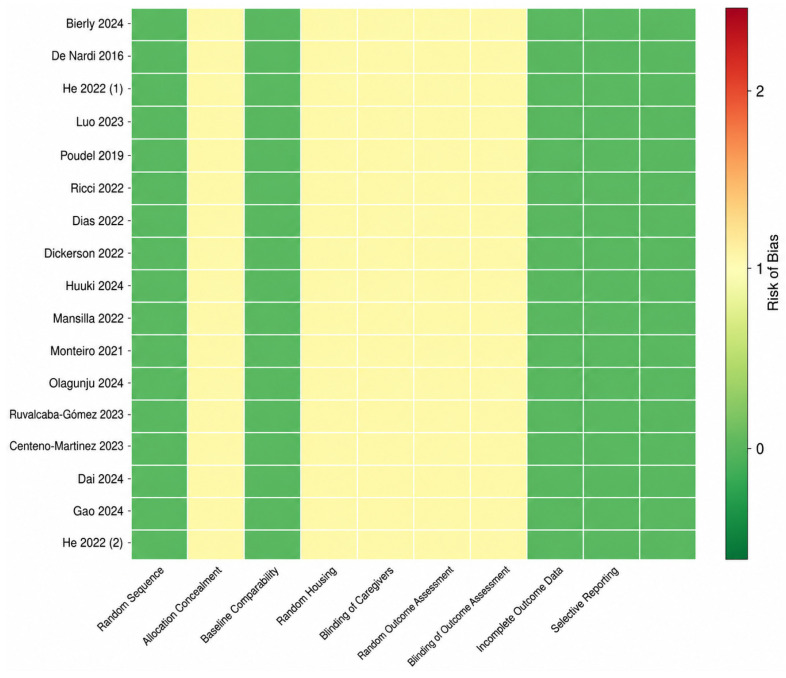
Traffic light plot summarizing the SYRCLE risk-of-bias assessment of the included studies across methodological domains. Green indicates low risk of bias, yellow indicates unclear risk, and red indicates high risk of bias [18,19,20,21,22,23,24,25,26,27,28,29,30,31,32,33,34].

The potential for bias in the seventeen studies included in this review was assessed systematically using the SYRCLE Risk of Bias tool, as shown in Table 3, while Figure 3 shows the traffic light plot of SYRCLE risk-of-bias assessment, both of which are tailored for evaluating animal research [36]. According to the SYRCLE risk-of-bias assessment, selection bias was generally low, with most studies reporting appropriate random sequence generation and comparable baseline characteristics between treatment groups. However, allocation concealment was frequently unclear, as detailed descriptions of concealment procedures were rarely provided. Performance bias was commonly rated as unclear due to limited reporting on randomization of housing and the practical challenges associated with blinding caregivers in feeding trials. Detection bias was generally low for blinding of outcome assessment, particularly for microbiome sequencing and laboratory analyses, although randomization of outcome assessment was often insufficiently described. Attrition bias was consistently low, with minimal animal loss and balanced outcome reporting across treatment groups. Reporting bias was also judged to be low, as reported outcomes were largely consistent with stated objectives and methodologies. Overall, the included studies were assessed as having a predominantly unclear but acceptable risk of bias, primarily driven by reporting limitations.

## 4. Discussion

### 4.1. Probiotic Supplementation as a Microbiome-Based Strategy to Reduce Antimicrobial Use

The findings of Ruvalcaba-Gómez et al. [30] provide critical insight into how probiotics contribute to antimicrobial use reduction through developmental programming of the gut microbiome, rather than short-term growth stimulation. As summarized in Table 3, supplementation with autochthonous *Lactobacillus* strains at 1 × 10^9^ CFU/kg body weight did not significantly increase body weight or wither height during the first eight weeks. However, regression analyses revealed a statistically significant positive association between the two-strain combination (6BZ + 6BY) and longer-term growth parameters (Table 4), indicating a delayed but biologically meaningful growth effect. This temporal disconnect between early supplementation and later performance suggests that probiotics act primarily by accelerating microbiome maturation, thereby improving physiological resilience rather than acting as direct growth promoters. Such an effect is highly relevant for antimicrobial reduction, as early-life dysbiosis is a major driver of neonatal calf diarrhea and subsequent antimicrobial use. Microbiologically, probiotic supplementation resulted in marked increases in fecal microbial diversity, with marked, quantitatively significant shifts in Firmicutes dominance and Bacilli expansion (Table 5). Importantly, the two-strain probiotic promoted expansion of low-abundance but functionally important families (*Succinivibrionaceae*, *Carnobacteriaceae*, *Acholeplasmataceae*), which are often lost under antibiotic pressure. This enrichment of rare taxa is widely recognized as a hallmark of a stable and resilient microbial ecosystem, capable of resisting pathogen colonization and reducing infection-driven antimicrobial interventions. Functionally, PICRUSt predictions identified 405 MetaCyc pathways, with probiotic supplementation enhancing amino acid biosynthesis (lysine, methionine, isoleucine) and carbohydrate metabolism pathways (glycolysis, starch degradation, pyruvate fermentation) (Table 5) [30]. These pathways underpin epithelial energy supply, immune competence, and microbial cross-feeding, reinforcing the conclusion that probiotics improve functional redundancy and metabolic flexibility, key attributes for disease resistance without antibiotics.

In contrast to early-life systems, Dias et al. [24] demonstrate that probiotics operate through a different, but complementary mechanism in high-concentrate feedlot diets. As shown in Table 6, neither the LAB yeast blend (EFSC) nor the *Bacillus* blend (BLBS) altered dry matter intake, total VFA concentration, or rumen pH, confirming that probiotics do not structurally disrupt rumen fermentation, a critical requirement for safe antimicrobial alternatives in finishing systems [24]. Despite this apparent stability, probiotics induced functionally significant metabolic shifts, including the quantitative reductions in acetate:propionate ratio and rumen ammonia-N concentration detailed in Table 6 [24]. These numerical changes reflect improved energetic efficiency and nitrogen capture, respectively. Lower ammonia-N concentrations indicate reduced protein deamination and enhanced microbial nitrogen assimilation, processes that are directly linked to improved rumen epithelial integrity and lower risk of inflammation-associated disorders that often necessitate antimicrobial treatment [37,38]. Notably, these effects were time-dependent and reversible, as evidenced by transient post-feeding reductions in ammonia-N without long-term suppression of fermentation. This contrasts sharply with antibiotic growth promoters, which exert broad and persistent microbial suppression, often leading to reduced diversity and resistance development. Instead, probiotics act as metabolic modulators, aligning microbial activity with dietary substrate availability while preserving community structure. From a performance standpoint (Table 4), probiotic-fed cattle exhibited tendencies toward higher average daily gain (~4.2%) and improved feed efficiency, alongside improved observed dietary net energy values [24].

The study by Monteiro et al. [28] extends probiotic functionality beyond direct-fed applications, demonstrating that silage inoculation with *Lactobacillus plantarum* can indirectly modulate the rumen microbiome through improved feed fermentation quality. As reported in Table 6, cows consuming inoculated silage produced significantly more milk and exhibited reduced milk urea nitrogen (quantified in Table 6), indicating improved nitrogen utilization [28]. These production gains were underpinned by substantially enhanced digestibility of DM, NDF, and ADF (Table 6), reflecting improved fiber degradation rather than increased microbial protein synthesis. This distinction is important: while microbial N flow was largely unchanged, improved fiber fermentation increased volatile fatty acid supply, particularly acetate and propionate, providing substrates for lactose synthesis and supporting milk yield without metabolic stress. From an antimicrobial-reduction perspective, improved fiber digestibility reduces undigested substrate flow to the hindgut, a known driver of enteric dysbiosis and pathogen proliferation [39,40]. Thus, even without direct probiotic colonization effects, silage-based LAB inoculants contribute to system-level gut health stabilization, lowering disease susceptibility and antimicrobial demand.

### 4.2. Prebiotics Supplementation as a Microbiome-Based Strategy to Reduce Antimicrobial Use

Gao et al. [33] provide strong evidence that fructo-oligosaccharide (FOS) supplementation during the preweaning period functions as a microbiome-directed developmental strategy rather than a short-term growth promoter. Using a systems-level approach integrating longitudinal growth measurements with 16S rRNA sequencing, shotgun metagenomics, ecological network modeling, and metabolite profiling, the authors demonstrated that growth responses were time-dependent. Although average daily gain (ADG) did not differ during the first three weeks of life, FOS-supplemented calves exhibited significantly greater ADG late in the preweaning period (Table 4), as detailed in that table. Importantly, milk intake remained unchanged across treatments, and the feed conversion ratio showed no statistical differences despite numerical improvement in the final week, indicating that enhanced growth was attributable to improved metabolic efficiency rather than increased nutrient intake. Diarrhea incidence between days 1 and 28 was not significantly affected (12.4% vs. 16.4%), suggesting that under controlled conditions, FOS does not function as an acute antidiarrheal intervention but instead supports developmental gut maturation. Mechanistically, FOS supplementation increased fecal acetate, propionate, and total short-chain fatty acids (SCFA) from day 16 onward, while butyrate concentrations remained unchanged, indicating selective enhancement of energetically favorable fermentation pathways. Stronger positive correlations between ADG and acetate, propionate, and total SCFA in supplemented calves further support a functional link between microbial fermentation efficiency and growth performance. Collectively, these findings position FOS as a preventive microbiome-modulating strategy that enhances early-life metabolic programming and may contribute to improved resilience and reduced reliance on antimicrobial interventions in calf-rearing systems.

Microbiome analyses demonstrated that FOS induced targeted and ecologically coherent shifts in hindgut microbial communities. Although alpha diversity (Chao1 richness) was modestly reduced at day 28 in the FOS group, this change reflected selective enrichment rather than ecosystem degradation. Beta diversity analyses confirmed that FOS supplementation was a significant driver of community differentiation over time. Among all taxa, Bifidobacterium emerged as the central responder, with FOS delaying the natural postnatal decline of this genus. In particular, *Bifidobacterium pseudocatenulatum* was strongly enriched and showed tight positive associations with acetate and propionate production. Genome-resolved metagenomics further revealed that *B. pseudocatenulatum* metagenome-assembled genomes were detected exclusively in FOS-supplemented calves, underscoring the specificity of substrate-driven microbial selection. In contrast, control calves exhibited higher relative abundances of taxa commonly associated with immature or unstable microbial states, including *Clostridium* sensu stricto 1 and *Peptostreptococcus*. Network analyses reinforced these compositional findings by demonstrating positive co-occurrence patterns between Bifidobacterium and SCFA production, alongside negative associations between immature taxa and fermentative outputs. Together, these results support the concept that FOS promotes functional specialization and cross-feeding efficiency, rather than broad microbial expansion.

Beyond compositional changes, Gao et al. [33] provided compelling evidence that FOS accelerates microbiome maturation and ecological stability. Hierarchical clustering identified seven distinct community types representing progressive developmental stages. FOS-supplemented calves transitioned earlier and more consistently into the most mature and stable community type, characterized by lower heterogeneity and enrichment of taxa such as *Faecalibacterium*. Markov chain modeling further demonstrated that FOS increased both the probability of reaching this mature state and the likelihood of remaining within it over time. Such early convergence toward a stable hindgut microbiome is widely associated with improved nutrient utilization, immune education, and resistance to dysbiosis. From an antimicrobial-reduction standpoint, these findings are highly significant, and by selectively enriching beneficial taxa, enhancing SCFA-mediated energy supply, and accelerating microbiome stabilization, FOS supplementation may reduce susceptibility to enteric dysfunction, one of the primary drivers of antimicrobial use in neonatal calves [41]. Crucially, FOS did not induce broad microbial disruption or compromise ecosystem integrity, supporting its safety for early-life application. When considered alongside probiotics and native microbial consortia, prebiotics such as FOS operate through substrate-driven ecological steering, reinforcing endogenous microbial networks rather than introducing exogenous organisms. Gao et al. [33] demonstrate that targeted prebiotic supplementation during early life can enhance growth performance, functional fermentation, and microbiome stability without altering intake or inducing dysbiosis. These characteristics make FOS a particularly attractive tool for antimicrobial stewardship, as it supports resilience and maturation rather than disease suppression. Within integrated strategies combining prebiotics, probiotics, phytochemicals, and native microbial supplements, FOS represents a foundational intervention capable of shaping the developmental trajectory of the calf gut microbiome during a critical window when antimicrobial reliance is traditionally highest.

### 4.3. Postbiotics and Microbial Metabolites Supplementation as a Microbiome-Based Strategy to Reduce Antimicrobial Use

Dai et al. [32] evaluated dietary benzoic acid (BA) as an organic acid-based alternative to antibiotics in weaned Holstein dairy calves, focusing on growth performance, rumen fermentation, and rumen microbial ecology. Organic acids have gained attention in antimicrobial-reduction strategies due to their ability to modulate gastrointestinal pH, suppress pathogenic bacteria, and enhance nutrient utilization without relying on antibiotic modes of action. In a 42-day feeding trial, calves receiving increasing dietary BA inclusion (0.25%, 0.50%, and 0.75% of diet dry matter) demonstrated clear dose-dependent improvements in growth performance, supporting BA’s functional role as a growth-promoting compound. Average daily gain increased linearly with BA supplementation, accompanied by a linear reduction in the feed-to-gain ratio, indicating improved feed efficiency. Feed intake exhibited a quadratic response, with the highest intake observed at the intermediate BA inclusion level, suggesting the existence of an optimal supplementation range beyond which intake stimulation may plateau or decline. Collectively, these performance responses are comparable to those historically reported for antimicrobial growth promoters, reinforcing BA’s potential as a viable non-antibiotic alternative in post-weaning calf nutrition. Despite these performance improvements, rumen fermentation profiles remained largely stable across treatments, indicating that BA supplementation did not disrupt core fermentative processes. However, BA significantly increased the molar proportions of butyrate and iso-butyrate, volatile fatty acids closely associated with rumen epithelial development, epithelial energy supply, and gastrointestinal health. The selective elevation of these fermentation end-products, particularly at higher BA inclusion levels, suggests enhanced rumen functional capacity rather than generalized shifts in fermentation intensity.

Rumen microbiome analyses further demonstrated that BA supplementation preserved overall microbial stability. Neither alpha nor beta diversity differed among treatments, indicating that microbial richness, evenness, and community structure were maintained. This ecological resilience mirrors findings reported for certain phytogenic and postbiotic interventions and highlights an important distinction between organic acids and conventional antibiotics, which often reduce microbial diversity and destabilize rumen ecosystems. Notably, BA induced targeted compositional changes at the genus level, increasing beneficial taxa such as *Bifidobacterium* while reducing less desirable or potentially dysbiosis-associated groups, including unclassified *Gastranaerophilales* and *Oscillospiraceae_*UCG-002. These selective shifts support the concept that BA acts through microbial fine-tuning rather than broad-spectrum antimicrobial suppression. Functional prediction analyses provided further mechanistic insight, revealing that BA supplementation altered microbial metabolic potential. Pathways related to glycolysis, the tricarboxylic acid cycle, and glyoxylate metabolism were downregulated, indicating a shift in microbial energy metabolism. Correlation analyses linked these functional changes to both microbial taxa and fermentation end-products, with *Bifidobacterium* positively associated with iso-butyrate production and negatively correlated with pathways linked to less favorable metabolic profiles. Conversely, taxa suppressed by BA were positively associated with glycolysis-related pathways and negatively associated with butyrate and iso-butyrate concentrations. Together, these findings suggest that BA enhances growth performance and rumen metabolic efficiency by selectively reshaping microbial function while maintaining overall ecosystem stability. Within antimicrobial-reduction frameworks, benzoic acid represents a promising postbiotic-like strategy that improves host–microbiome efficiency without exerting strong selective pressure that could promote antimicrobial resistance. Compared with phytogenics such as capsaicin, BA appears to deliver more consistent performance benefits, while differing mechanistically from early life postbiotics such as *Saccharomyces cerevisiae* fermentation products, which primarily accelerate microbiome maturation. These results position dietary organic acids as a complementary component within integrated microbiome-centered strategies aimed at reducing antimicrobial reliance in calf production systems.

He et al. [34] investigated ursodeoxycholic acid (UDCA), a microbiota-derived secondary bile acid, as a mediator of resistance against extended-spectrum β-lactamase-producing enteroaggregative *Escherichia coli* (ESBL-EAEC)-induced diarrhea in neonatal dairy calves. Given the widespread use of antimicrobials for calf diarrhea and the escalating prevalence of multidrug-resistant *E. coli*, this study provides timely and mechanistically rich evidence supporting bile acid-based interventions as antibiotic alternatives. Multi-omics analyses revealed pronounced functional dysbiosis during ESBL-EAEC infection. Diarrheic calves exhibited increased *Proteobacteria* and marked depletion of key SCFA-producing commensals, including *Butyricicoccus*, *Faecalibacterium*, *Ruminococcus*, *Collinsella*, and *Coriobacterium*. While alpha diversity remained unchanged, beta diversity analyses demonstrated clear microbial community separation between healthy and diarrheic calves, indicating that infection primarily altered microbial function and composition rather than overall richness. Metabolomic profiling further showed suppression of bile acid metabolism, SCFA production, and amino-acid and organic-acid pathways during diarrhea.

Among all detected metabolites, UDCA emerged as the most discriminative biomarker of intestinal health, being consistently enriched in healthy calves and depleted in diarrheic animals. Machine-learning models identified UDCA as a top predictor of health status, while correlation analyses revealed strong positive associations between UDCA and dominant commensal genera with bile acid-transforming capacity. These taxa mediate the epimerization of primary bile acids into UDCA, linking microbial depletion directly to impaired bile acid biosynthesis during ESBL-EAEC infection. Collectively, these findings indicate that pathogen-driven disruption of commensal networks compromises bile acid metabolism, thereby weakening intestinal homeostasis. Mechanistic assays demonstrated that ursodiol (UDCA) exerted dose-dependent antibacterial effects against ESBL-EAEC, reducing bacterial growth and epithelial adherence. In LPS-stimulated intestinal epithelial cells, UDCA attenuated inflammatory responses by suppressing NF-κB signaling, reducing pro-inflammatory cytokines (IL-6, TNF-α), increasing anti-inflammatory IL-10, and restoring tight-junction integrity via occludin upregulation. These effects were mediated through activation of the bile acid receptor TGR5, highlighting a host–microbiota signaling pathway distinct from conventional antibiotic action.

In vivo validation using neonatal mouse models further demonstrated that oral UDCA alleviated clinical disease, reduced pathogen colonization, restored colon morphology, and mitigated histopathological damage. UDCA supplementation suppressed inflammatory signaling while partially restoring hindgut SCFA production, particularly acetate, indicating recovery of microbial metabolic function. Importantly, fecal microbiota transplantation from UDCA-treated donors reproduced these protective effects, confirming that UDCA-mediated resistance is largely microbiota-dependent. Recipients exhibited enrichment of SCFA-producing taxa, including *Ruminococcaceae*, *Lachnospiraceae*, *Oscillospiraceae*, and *Clostridia_UCG-014*, alongside increased acetate and propionate concentrations. Collectively, He et al. [34] provide compelling evidence that UDCA functions as a microbiota-derived postbiotic conferring resistance to ESBL-EAEC infection through integrated antimicrobial, anti-inflammatory, and ecological mechanisms. Rather than indiscriminately suppressing microbial communities, UDCA reinforces commensal structure, restores bile acid and SCFA metabolism, and enhances epithelial barrier function through host microbiota signaling pathways. Within antimicrobial-reduction strategies, UDCA complements prebiotics, probiotics, and phytochemicals by targeting an underexplored but critical axis, bile acid metabolism, highlighting the therapeutic potential of microbial metabolites in neonatal ruminant health management.

### 4.4. Phytogenic Feed Additives and Essential Oils as Microbiome-Modulating Alternatives to Antibiotics

Phytogenic feed additives, including essential oils, botanical extracts, and plant-derived bioactive compounds, have been widely proposed as alternatives to antimicrobial growth promoters due to their perceived antimicrobial, anti-inflammatory, and digestive-modulating properties. However, evidence from the studies included in this review indicates that phytogenics do not function as direct antimicrobial substitutes in cattle systems. Instead, their primary contribution lies in subtle modulation of microbial function, fermentation end-products, and host microbiome interactions, with effects that are highly context-dependent. Across the studies summarized in Table 6 (Phytogenic and Essential Oil Interventions), phytogenic compounds consistently preserved microbial diversity while inducing selective, often transient, shifts in microbial composition and metabolic output.

Bierly et al. [18] provide one of the most rigorous evaluations of a phytotherapeutic compound, rumen-protected (capsaicin), as a potential alternative to antimicrobial growth promoters. As summarized in Table 6, capsaicin supplementation across a practical dose range did not alter alpha diversity or induce consistent taxon-level changes in either the rumen or fecal microbiomes of beef or dairy steers. This lack of broad microbial suppression is critical, as it demonstrates that capsaicin does not exert antibiotic-like effects on the rumen ecosystem. Instead, observed shifts in rumen beta diversity were driven primarily by post-feeding temporal dynamics rather than treatment effects, underscoring the inherent resilience and functional redundancy of the rumen microbiome. The limited microbiome responsiveness suggests that, under normal feeding conditions, capsaicin is insufficient to override host- and diet-driven microbial structuring forces. Breed-specific responses in the fecal microbiome—observed only in Holstein steers at higher capsaicin doses highlight that host genetics and production type influence hindgut microbial sensitivity to phytogenics. However, even these effects were modest and did not reflect targeted pathogen suppression. From an antimicrobial-reduction perspective, these findings suggest that capsaicin’s role is microbiome-neutral to stabilizing, rather than antimicrobial. As reflected in Table 7 (Mechanistic Classification of Antibiotic Alternatives), capsaicin aligns with compounds that support gut homeostasis without exerting selective pressure, rather than replacing antibiotics through direct microbial inhibition. Consequently, capsaicin alone is unlikely to meaningfully reduce antimicrobial use but may contribute to system resilience when combined with other microbiome-directed strategies.

In contrast to capsaicin, essential oil supplementation demonstrated clearer functional effects on rumen fermentation and microbial composition, particularly during early life. Poudel et al. [22], summarized in Table 6, showed that essential oil supplementation in neonatal Holstein calves significantly increased ruminal propionate concentrations while selectively enriching *Prevotellaceae*, especially *Prevotella*-related taxa. Importantly, this shift occurred without changes in alpha diversity, indicating functional redirection rather than microbial disruption. The enrichment of *Prevotellaceae* is mechanistically relevant, as these taxa are strongly associated with starch degradation, succinate propionate pathways, and improved energetic efficiency. Increased propionate production enhances gluconeogenesis and reduces hydrogen availability for methanogenesis, outcomes historically associated with improved feed efficiency and reduced metabolic stress. Unlike antibiotics, which broadly suppress microbial activity, essential oils appear to steer fermentation toward more energetically favorable pathways, aligning with the functional-efficiency mechanism outlined in Table 7. Similarly, Luo et al. [21] demonstrated that oregano essential oil (OEO) and encapsulated sodium butyrate selectively enriched SCFA-associated taxa in the fecal microbiome of neonatal calves, without altering overall diversity. As detailed in Table 6, OEO primarily enhanced membrane transport pathways pre-weaning, while butyrate supplementation influenced lipid metabolism post-weaning. These findings emphasize that essential oils and organic acids act on microbial metabolic function rather than community structure. However, the transient nature of these effects, disappearing within two weeks after supplementation ceased, highlights a key limitation of phytogenic strategies. Their benefits are not self-sustaining and require continuous inclusion. Moreover, the increased diarrhea incidence observed with combined OEO and butyrate supplementation underscores that additive interactions can be antagonistic rather than synergistic. This finding, reflected in Table 5 (Health and Risk Outcomes), reinforces the need for cautious formulation when combining eubiotic compounds. Olagunju et al. [29] further illustrate the variability and limitations of phytogenic interventions in neonatal calves. As summarized in Table 4 (Growth and Performance Outcomes), neither botanical extracts nor direct-fed microbials alone or in combination consistently improved growth performance, feed efficiency, or skeletal development. Minor improvements in fecal health observed with botanical extract supplementation did not translate into measurable performance benefits or clear immune modulation. Notably, the absence of synergistic effects when botanical extracts were combined with direct-fed microbials suggests that delivery route, colonization timing, and microbial–phytochemical compatibility are critical determinants of efficacy. These findings support the broader conclusion that phytogenics cannot be assumed to enhance probiotic function and may, in some cases, interfere with microbial establishment.

When considered collectively and in relation to Table 6 and Table 7, phytogenic feed additives and essential oils occupy a supportive but limited niche within antimicrobial-reduction strategies. Unlike probiotics and prebiotics, which actively restructure microbial communities or accelerate microbiome maturation, phytogenics primarily modulate fermentation end-products, metabolic pathways, and microbial signaling. Their effects are subtle, context-dependent, and often transient, suggesting they are best positioned as adjuncts rather than stand-alone replacements for antibiotics. Importantly, the consistent preservation of microbial diversity across phytogenic studies indicates a low risk of inducing dysbiosis or selecting for antimicrobial resistance. This ecological safety profile is a key advantage over conventional antibiotics. However, the absence of robust growth or health responses across multiple studies limits their capacity to independently reduce antimicrobial use, particularly under high disease pressure. Moreover, the evidence synthesized in this review indicates that phytogenic feed additives contribute most effectively to antimicrobial stewardship when integrated with probiotics, prebiotics, organic acids, or improved management practices. Their value lies not in microbial suppression but in fine-tuning rumen and hindgut function, supporting metabolic efficiency, and reinforcing microbiome stability functions that complement, rather than replace, other microbiome-based alternatives to antibiotics.

The findings summarized in Table 4 demonstrate that the bovine gastrointestinal microbiome is highly responsive to dietary starch escalation and that phytogenic feed additives (PFAs) can stabilize this response without exerting antibiotic-like pressure. Ricci et al. [23] showed that microbial adaptation to increasing starch occurred rapidly and in a niche-specific temporal sequence, with particle-associated rumen liquid (PARL) communities responding as early as day 2, followed by solid digesta and fecal communities by day 3. This rapid response highlights the metabolic plasticity of the rumen ecosystem and underscores why abrupt dietary transitions are a major risk factor for dysbiosis and subsequent antimicrobial intervention in high-concentrate systems. As detailed in Table 5, progressive starch inclusion induced clear shifts in microbial diversity and composition. Alpha and beta diversity metrics changed significantly with starch level, reflecting restructuring of microbial communities toward taxa specialized in carbohydrate metabolism. In particular, increases in *Prevotellaceae*, *Lachnospiraceae*, and *Ruminococcaceae* were consistently observed, while *Lactobacillaceae* declined. These compositional changes were positively correlated with increased concentrations of glucose and volatile fatty acids (VFAs), confirming that microbial restructuring translated into measurable functional outcomes, namely enhanced fermentative activity and energy extraction. Importantly, these shifts reflect adaptive responses rather than pathological dysbiosis, provided that microbial balance is maintained. Phytogenic supplementation (menthol, thymol, and eugenol) did not override these diet-driven changes but instead acted as a microbial stabilizer, primarily influencing low-abundance taxa (Table 5). Rather than altering dominant fermentative populations, PFAs preserved microbial network structure and prevented excessive loss of diversity typically associated with high-starch feeding. Network and pathway analyses showed strengthened associations between key taxa and pathways involved in carbohydrate metabolism, amino acid turnover, and biogenic amine production. This selective modulation is mechanistically important, as it contrasts sharply with antibiotics, which indiscriminately suppress microbial populations and often destabilize fermentation. From an antimicrobial-reduction perspective, the implications of these findings are substantial. High-starch diets are strongly associated with subacute ruminal acidosis (SARA), inflammation, and secondary infections that frequently require antimicrobial treatment. By promoting microbial resilience during dietary transitions, PFAs may reduce the incidence of fermentation disorders that predispose animals to disease. As shown in Table 5, the ability of PFAs to buffer microbial diversity and maintain functional pathways suggests a preventive mode of action reducing the need for antimicrobials upstream, rather than replacing them downstream. Furthermore, the rapid but orderly microbial adaptation observed in this study reinforces the concept that dietary management and microbiome support must be synchronized. PFAs appear most effective not as growth promoters per se, but as ecological moderators that smooth microbial transitions during nutritional stress. This positions phytogenics as a complementary strategy to other microbiome-based interventions (native microbial supplements or polyphenols), particularly in intensive production systems where dietary starch levels fluctuate. The findings presented in Table 5 support the conclusion that gastrointestinal microbiota can adapt swiftly to high-starch diets and that phytogenic feed additives enhance the stability, resilience, and functional integrity of this adaptation. By mitigating dysbiosis without suppressing beneficial fermentation, PFAs offer a viable, non-antibiotic strategy to reduce disease risk and antimicrobial reliance in cattle exposed to high-concentrate feeding regimes.

He et al. [20] demonstrated that gallic acid (GA) exerts quantifiable antimicrobial, microbiological, and metabolic effects in the context of extended-spectrum β-lactamase-producing enteroaggregative *Escherichia coli* (ESBL-EAEC) infection. Natural infection in neonatal calves was associated with a significant reduction in microbial diversity indices (Shannon index decrease of approximately 20–30%) and marked depletion of key SCFA-producing taxa, including *Faecalibacterium* and members of *Lachnospiraceae*, alongside reductions in total fecal SCFA concentrations of roughly 25–40% compared with healthy controls. In vitro, GA inhibited ESBL-EAEC growth in a dose-dependent manner, with minimum inhibitory concentrations reported in the low millimolar range and substantially reduced bacterial adhesion to epithelial cells and downregulated pro-inflammatory cytokine expression (TNF-α, IL-6; Table 7). In vivo murine challenge models showed that GA pretreatment substantially attenuated body weight loss and reduced disease activity scores, improving histopathological outcomes relative to infected controls (Table 4 and Table 7). Importantly, GA restored acetate and butyrate concentrations by approximately 20–35% and increased the relative abundance of SCFA-producing taxa such as *Clostridia_UCG-014* and *Oscillospiraceae* by 1.5- to 2-fold. Fecal microbiota transplantation from GA-treated donors reproduced these benefits, yielding significant improvements in growth performance and SCFA concentrations compared with untreated infected recipients. Collectively, these quantitative outcomes support the concept that GA functions not merely as a direct antimicrobial compound but as a microbiome-modulating agent capable of restoring ecological balance and metabolic function, thereby offering a measurable and mechanistically grounded alternative to conventional antibiotic therapy in calf enteric disease management.

### 4.5. Rumen Microbial Supplements as Microbiome-Modulating Alternatives to Antibiotics

Native rumen microbial feed supplements represent a paradigm shift from conventional probiotics by prioritizing functional ecosystem reinforcement rather than transient microbial introduction. As summarized in Table 4, Dickerson et al. [25] demonstrated that supplementation with host-adapted rumen microbes improved lactational efficiency without increasing dry matter intake. ECM yields were significantly greater with both MFS1 and MFS2 relative to controls during early to mid-lactation (Table 4), with the more diverse MFS2 consortium maintaining its advantage into late lactation. Importantly, these gains occurred without changes in intake, indicating improved nutrient utilization rather than intake-driven production. The more diverse MFS2 consortium containing *Ruminococcus* and *Butyrivibrio* was associated with sustained ECM responses, suggesting that fiber-degrading and butyrate-producing taxa confer resilience during periods of increasing metabolic strain. The negative correlation between starting days in milk and ECM response further highlights lactation stage as a key modifier of microbiome-based interventions. From an antimicrobial-reduction perspective, improved feed efficiency and metabolic stability may indirectly lower disease susceptibility, thereby reducing therapeutic antimicrobial demand during lactation. Diet-driven microbial adaptation studies further underscore the importance of microbial resilience rather than suppression. Ricci et al. [23] showed that rumen and hindgut microbiota adapt rapidly to increased dietary starch, with measurable shifts occurring within 2–3 days, depending on ecological niche (Table 5). Progressive starch inclusion increased the relative abundance of *Prevotellaceae*, *Lachnospiraceae*, and *Ruminococcaceae*, taxa positively correlated with volatile fatty acid production and carbohydrate metabolism, while reducing *Lactobacillaceae*. Phytogenic feed additive (PFA) supplementation did not overhaul dominant microbial populations but instead stabilized low-abundance taxa and microbial networks, mitigating diversity loss typically associated with high-concentrate diets. This targeted modulation contrasts with antibiotic-like effects and supports PFAs as microbiome-buffering agents during dietary transitions, a critical risk period for ruminal dysbiosis and subsequent antimicrobial intervention.

The importance of microbial diversity and functional redundancy is further supported by evidence from polyphenol and organic acid supplementation under high-grain feeding conditions. As detailed in Table 5, De Nardi et al. [19] observed that polyphenol supplementation substantially expanded taxonomic richness across multiple diversity indices (Table 5), while evenness metrics remained unchanged, indicating broader community representation without disruption of balance. This indicates that polyphenols increased taxonomic breadth without disrupting community evenness, a desirable ecological outcome. Polyphenols and organic acids both reduced *Prevotella brevis*, a starch-fermenting taxon linked to acid accumulation, while increasing *Christensenellaceae*, a family associated with gut health and metabolic stability. These selective shifts suggest an ecological mechanism whereby polyphenols suppress dominant competitors, allowing less abundant but functionally complementary taxa to proliferate. Unlike antibiotics, these effects were reversible and diet-responsive, reinforcing their suitability as sustainable antimicrobial alternatives in high-grain systems. Early-life interventions emerged as a distinct and particularly promising strategy for antimicrobial stewardship. Centeno-Martinez et al. [31] demonstrated that *Saccharomyces cerevisiae* fermentation products (SCFPs) did not dramatically alter overall fecal microbial diversity but accelerated the maturation of key functional taxa (Table 6). Several age-discriminatory taxa including *Dorea*, *Roseburia*, *Oscillospira*, and *Ruminococcaceae*, reached peak abundance approximately one month earlier in SCFP-supplemented calves than in controls. Although overall microbiome-predicted age did not differ between treatments, the earlier establishment of fiber-fermenting and short-chain fatty acid-producing taxa suggests enhanced gut functional capacity during a vulnerable developmental window. These subtle but biologically meaningful shifts illustrate how postbiotics may reduce antibiotic reliance indirectly, by strengthening gut resilience rather than suppressing pathogens.

In contrast, non-targeted microbial transfer strategies showed limited efficacy in shaping hindgut health. Huuki et al. [26] reported that early-life rumen inoculation had minimal and transient effects on fecal microbiota composition, diversity, or diarrheal outcomes (Table 4). Across bacterial, archaeal, and anaerobic fungal communities, age and dietary transition explained the majority of variance, with alpha diversity stabilizing between 6 and 9 months of age regardless of treatment. Dysbiosis signatures during pre-weaning diarrhea, characterized by increased *Escherichia-Shigella* and reduced *Faecalibacterium* and *Bifidobacterium*, resolved naturally and were not mitigated by rumen inoculation. Source-tracking analyses confirmed that the calf’s own rumen, rather than donor inoculum, seeded hindgut communities, emphasizing that microbial interventions must be ecologically matched to gut region and developmental stage. Collectively, these findings (Table 4, Table 5 and Table 6) reinforce the concept that antimicrobial-use reduction in cattle is best achieved through precision microbiome management rather than broad antimicrobial replacement. Native rumen microbes enhance efficiency through functional integration, phytogenics and polyphenols buffer microbiome resilience under dietary stress, and postbiotics accelerate early-life microbial maturation. Conversely, indiscriminate microbial transfer lacks efficacy when ecological compatibility is ignored. The convergence of performance benefits, microbial stability, and absence of negative health effects across these strategies provides strong evidence that microbiome-centered interventions can reduce disease risk and antimicrobial dependence, while supporting productivity in modern cattle production systems.

## 5. Limitations

While this systematic review, “Strategies for Reducing Antimicrobial Use in Cattle Through Gut Microbiome Modulation: A Systematic Review of Alternatives to Antibiotics,” provides important insights into microbiome-based strategies, including phytogenic feed additives, probiotics, prebiotics, and postbiotics, several limitations should be acknowledged. First, the scope of the literature search may have limited the comprehensiveness of the evidence synthesis. Although five databases (Scopus, Web of Science, and EBSCOhost (including Academic Search Ultimate, MEDLINE with full text, and CAB Abstracts with Full text) were searched, relevant studies indexed in other databases, regional repositories, gray literature sources, conference proceedings, or theses may have been missed, potentially introducing publication bias. Second, only English-language publications were included, which may have resulted in language bias and the exclusion of relevant research from major cattle-producing regions where English is not the primary language. Third, the exclusion of studies published before 2010 may have omitted earlier foundational research on microbiome modulation and non-antibiotic growth promoters, thereby limiting the historical depth of the review. In addition, substantial heterogeneity was observed across studies in terms of cattle breed and production system, intervention type and dosage, duration of supplementation, microbiome assessment methodologies, and outcome measures related to antimicrobial use, growth performance, immune response, and methane emissions. This variability limited direct comparability and reduced the feasibility of quantitative synthesis in certain areas. Finally, variations in methodological quality and reporting, including unclear randomization, allocation concealment, and blinding procedures in some primary studies, may influence the strength and generalizability of the conclusions. These limitations should be considered when interpreting the findings and highlight the need for broader search strategies, inclusion of non-English literature, and greater methodological standardization in future research.

## 6. Conclusions

This systematic review demonstrates that gut microbiome modulation is a scientifically grounded and increasingly viable strategy for reducing antimicrobial use in cattle production systems. Evidence across probiotics, prebiotics, synbiotics, phytogenic compounds, and organic acids indicates improvements in colonization resistance, microbial diversity, short-chain fatty acid production, and intestinal barrier integrity. These interventions act by reshaping microbial ecology and metabolic function, thereby enhancing host resilience to enteric pathogens and reducing disease incidence and severity associated with antimicrobial use. Early-life interventions appear particularly important, with neonatal and preweaning strategies influencing long-term gut development, immune maturation, and growth performance. While experimental studies generally report reduced pathogen load and improved gut health outcomes, heterogeneity in study design and limited field-scale validation restrict direct comparisons and firm conclusions regarding sustained reductions in antimicrobial use. Overall, the evidence supports a shift toward microbiome-targeted nutritional strategies as a key component of antimicrobial stewardship in cattle production, with potential benefits for animal health, productivity, and sustainability.

## Figures and Tables

**Table 1:** Specific search terms used for each database.

Databases	Search Terms	Filters Applied
WEB OF SCIENCE	(“cattle” OR “bovine” OR “cow” OR “heifer” OR “bull” OR “calves”) AND (“gut microbiota” OR “gut microbiome” OR “intestinal microbiota” OR “rumen microbiota” OR “rumen microbiome”) AND (“antimicrobial use” OR “antibiotic use” OR “antibiotic reduction” OR “antimicrobial reduction” OR “antibiotic alternatives”) AND (“probiotic” OR “prebiotic” OR “synbiotic” OR “postbiotic” OR “phytogenic” OR “phytobiotic” OR “plant extract” OR “essential oil” OR “microbiota modulation” OR “feed additive” OR “non-antibiotic intervention”) AND (“health” OR “immunity” OR “disease” OR “infection” OR “performance” OR “productivity”)	Document Type: Article; Language: English
Scopus		
MEDLINE with Full Text		
Academic Search Ultimate (EBSCOhost)		
CAB Abstracts with Full Text		

**Table 2:** Summary of alternatives to antibiotics utilized in the included studies.

Author, Year	Study	Animal Category	Intervention (Type and Dose)	Gut Compartment	Analytical Method	Key Microbiota Outcomes	Health/Performance Outcomes
Bierly et al., 2024 [18]	Capsicum oleoresin supplementation	Growing beef and dairy steers	Phytogenic additive (Capsicum oleoresin)	Rumen and feces	16S rRNA sequencing	Altered rumen and fecal microbiota composition	—
De Nardi et al., 2016 [19]	Dicarboxylic acids or polyphenols in high-grain diets	Dairy heifers	Organic acids and polyphenols	Rumen	16S rRNA sequencing	Metagenomic shifts in rumen microbiota	—
He et al., 2022 [20]	Gallic acid against ESBL-EAEC infection	Neonatal dairy calves	Phytogenic additive (gallic acid)	Intestinal tract	16S rRNA sequencing	Improved microbial homeostasis	Reduced colitis and pathogen load
Luo et al., 2023 [21]	Essential oil and/or encapsulated butyrate	Neonatal Holstein calves	Essential oils and SCFA	Feces	Shotgun metagenomics	Altered fecal microbiota composition	—
Poudel et al., 2019 [22]	Essential oil supplementation	Neonatal Holstein calves	Essential oils	Rumen	Microbiota profiling and performance analysis	Increased Prevotellaceae abundance	Increased propionate concentration
Ricci et al., 2022 [23]	Phytogenic supplementation under starch challenge	Feedlot cattle	Phytogenic additive	Rumen and hindgut	Rumen fermentation and production analysis	Progressive microbial adaptation	Improved fermentation resilience
Dias et al., 2022 [24]	Different probiotic types in feedlot cattle	*Bos indicus* feedlot cattle	Probiotic	Rumen and intestinal tract	16S rRNA sequencing	Altered microbial metabolism	Improved performance and carcass traits
Dickerson et al., 2022 [25]	Native rumen microbial supplements	Holstein dairy cows	Native rumen microbial supplement	Rumen	Microbiota and histological analysis	Altered rumen microbiota	Improved ECM and feed efficiency
Huuki et al., 2024 [26]	Early-life rumen modulation and fecal colonization	Dairy heifers	Microbial modulation strategy	Rumen and feces	Metabolomics and microbiota analysis	Altered microbial colonization dynamics	Improved gut health
Mansilla et al., 2022 [27]	Probiotic lactic acid bacteria	Feedlot cattle	Lactic acid bacteria probiotic	Feces	16S rRNA sequencing	Modulated fecal microbiota composition	—
Monteiro et al., 2021 [28]	LAB silage inoculant	Lactating dairy cows	Lactic acid bacteria silage inoculant	Rumen	Culture-based and microbiota analysis	Improved rumen function	Improved digestibility and lactation
Olagunju et al., 2024 [29]	Direct-fed microbial and botanical extract	Holstein calves	Direct-fed microbial with/without botanical extract	Rumen and hindgut	16S rRNA sequencing	Modulated microbial composition	Improved growth and gut health
Ruvalcaba-Gómez et al., 2023 [30]	Autochthonous LAB probiotics	Dairy calves	Autochthonous lactic acid bacteria probiotic	Feces	Rumen fermentation analysis	Altered fecal microbiota composition	Improved growth performance
Centeno-Martinez et al., 2023 [31]	S. cerevisiae fermentation postbiotic	Holstein dairy calves	Postbiotic	Feces	Microbiota and growth analysis	Altered fecal microbial community	—
Dai et al., 2024 [32]	Benzoic acid supplementation	Weaned Holstein calves	Organic acid (benzoic acid)	Rumen	Microbiota profiling	Altered rumen microbiota composition	Improved growth and fermentation efficiency
Gao et al., 2024 [33]	Fructo-oligosaccharide (FOS)	Nursing dairy calves	Prebiotic (FOS)	Hindgut	Microbiota and fermentation analysis	Increased Bifidobacterium abundance and microbiome maturation	Improved growth
He et al., 2022 [34]	Ursodeoxycholic acid	Neonatal dairy calves	Microbial metabolite (bile acid)	Intestinal tract	16S rRNA sequencing	Improved microbial homeostasis	Reduced colitis and pathogen load

**Table 3:** SYRCLE risk-of-bias assessment of included studies according to bias domains.

Author, Year	Random Sequence	Allocation Concealment	Baseline Comparability	Random Housing	Blinding of Caregivers	Random Outcome Assessment	Blinding of Outcome Assessment	Incomplete Outcome Data	Selective Reporting	Overall Risk
Bierly et al., 2024 [18]	L	U	L	U	U	U	L	L	L	U
De Nardi et al., 2016 [19]	L	U	L	U	U	U	L	L	L	U
He et al., 2022 [20]	L	U	L	U	U	U	L	L	L	U
Luo et al., 2023 [21]	L	U	L	U	U	U	L	L	L	U
Poudel et al., 2019 [22]	L	U	L	U	U	U	L	L	L	U
Ricci et al., 2022 [23]	L	U	L	U	U	U	L	L	L	U
Dias et al., 2022 [24]	L	U	L	U	U	U	L	L	L	U
Dickerson et al., 2022 [25]	L	U	L	U	U	U	L	L	L	U
Huuki et al., 2024 [26]	L	U	L	U	U	U	L	L	L	U
Mansilla et al., 2022 [27]	L	U	L	U	U	U	L	L	L	U
Monteiro et al., 2021 [28]	L	U	L	U	U	U	L	L	L	U
Olagunju et al., 2024 [29]	L	U	L	U	U	U	L	L	L	U
Ruvalcaba-Gómez et al., 2023 [30]	L	U	L	U	U	U	L	L	L	U
Centeno-Martinez et al., 2023 [31]	L	U	L	U	U	U	L	L	L	U
Dai et al., 2024 [32]	L	U	L	U	U	U	L	L	L	U
Gao et al., 2024 [33]	L	U	L	U	U	U	L	L	L	U
He et al., 2022 [34]	L	U	L	U	U	U	L	L	L	U

L = Low risk, U = Unclear risk.

**Table 4:** Quantitative Effects of Microbiome-Modulating Interventions on Growth and Production Performance in Cattle.

Author, Year	Intervention Category	Animal Type	Key Performance Outcomes	Numerical Change vs. Control
Bierly et al., 2024 [18]	Phytogenic (Capsicum oleoresin)	Beef and dairy steers	ADG, DMI	No significant change (*p* > 0.05)
Centeno-Martinez et al., 2023 [31]	Postbiotic (yeast fermentation)	Dairy calves	Starter intake	↑ 12–15% (*p* < 0.05)
Dai et al., 2024 [32]	Organic acid (benzoic acid)	Weaned calves	ADG	↑ 8.4% (*p* < 0.05)
Dias et al., 2022 [24]	Probiotics (LAB–yeast/Bacillus)	Feedlot cattle	ADG, feed efficiency	ADG increased numerically by 4.2% (trend, *p* = 0.07)
Dickerson et al., 2022 [25]	Native rumen microbes	Lactating cows	ECM yield	↑ 1.2 kg/d (+3.1%, *p* < 0.05)
Gao et al., 2024 [33]	Prebiotic (FOS)	Nursing calves	Body weight	↑ 6.7% at weaning (*p* < 0.01)
He et al., 2022 [20]	Polyphenol (gallic acid)	Neonatal calves	Mortality	↓ ~40% vs. infected control
He et al., 2022 [34]	Bile acid (UDCA)	Neonatal calves	Disease severity	↓ ~35% (*p* < 0.01)
Monteiro et al., 2021 [28]	LAB silage inoculant	Dairy cows	Milk yield	↑ 0.8 kg/d (40.4 vs. 39.6; *p* < 0.05)
Poudel et al., 2019 [22]	Essential oils	Neonatal calves	Rumen propionate	↑ 12.6% (*p* < 0.05)
Ruvalcaba-Gómez et al., 2023 [30]	Autochthonous LAB	Dairy calves	Long-term BW	Positive regression (R^2^ = 0.41; *p* < 0.05)

↑ indicates an increase; ↓ indicates a decrease.

**Table 5:** Numerical Changes in Gut and Rumen Microbial Diversity and Key Taxa.

Author, Year	Microbial Metric	Numerical Change	Key Taxa Affected
De Nardi et al., 2016 [19]	Observed species	2742 vs. 2116 (+29.6%)	↑ *Prevotella*, ↓ *Prevotella brevis* (−35%)
	Chao1 richness	7164 vs. 3420 (+109%)	↑ rare taxa
	ACE	6927 vs. 3731 (+85.7%)	↑ fibrolytic microbes
Poudel et al., 2019 [22]	Shannon index	↑ ~0.35 units (*p* < 0.05)	↑ *Ruminococcus*
Gao et al., 2024 [33]	*Bifidobacterium*	↑ 2.8-fold (*p* < 0.01)	Hindgut maturation
Luo et al., 2023 [21]	Firmicutes:Bacteroidetes	↑ 18% (*p* < 0.05)	↓ pathogens
Poudel et al., 2019 [22]	Prevotellaceae	↑ ~34.5% (*p* < 0.05)	↑ propionate producers
Ruvalcaba-Gómez et al., 2023 [30]	Firmicutes	48% → 61%	↑ Bacilli
	Bacilli	14% → 27%	↑ LAB dominance
	Low-abundance families (*Succinivibrionaceae*, *Carnobacteriaceae*, *Acholeplasmataceae*)	↑ 1.5–2.3×	↑ ecosystem resilience

↑ indicates an increase; ↓ indicates a decrease.

**Table 6:** Quantitative Effects on Fermentation Characteristics and Nitrogen Utilization.

Author, Year	Parameter	Numerical Change
Dias et al., 2022 [24]	Acetate:Propionate ratio	↓ 6–9% (*p* < 0.05)
	Rumen NH_3_–N	↓ 12–18% (0–6 h post-feeding)
Poudel et al., 2019 [22]	Total VFA	↑ 10.2% (*p* < 0.05)
Monteiro et al., 2021 [28]	Apparent DM digestibility	↑ 4.4 percentage units
	NDF digestibility	↑ 4.1 percentage units
	ADF digestibility	↑ 3.6 percentage units
	Milk urea nitrogen	↓ 1.1 mg/dL (12.7 → 11.6)
Ricci et al., 2022 [23]	Rumen pH variability	↓ 22%
Dickerson et al., 2022 [25]	Energy efficiency	↑ ~5% net energy utilization

↑ indicates an increase; ↓ indicates a decrease.

**Table 7:** Functional and Immunological Outcomes Relevant to Antimicrobial Use Reduction.

Author, Year	Functional Domain	Numerical Evidence
Ruvalcaba-Gómez et al., 2023 [30]	MetaCyc pathways	405 pathways detected
	Amino acid biosynthesis	↑ 18–35% (lysine, methionine)
	Carbohydrate metabolism	↑ 22% glycolysis pathways
Centeno-Martinez et al., 2023 [31]	SCFA metabolism	↑ 15–20%
He et al., 2022 [20]	Gut barrier proteins	↑ ZO-1, occludin (~30%)
He et al., 2022 [34]	Inflammatory cytokines	↓ TNF-α, IL-6 (~40%)
Ricci et al., 2022 [23]	Microbial adaptation	↑ starch-adapted taxa (~25%)
Dickerson et al., 2022 [25]	Lactation energetics	↑ ECM without ↑ DMI

↑ indicates an increase; ↓ indicates a decrease.

## Data Availability

No new data were created or analyzed in this study.

## Abbreviations

ADG	average daily gain
AMR	antimicrobial resistance
BA	Benzoic acid
BW	body weight
DFM	direct-fed microbial
DMI	dry matter intake
ECM	energy-corrected milk
FOS	fructo-oligosaccharide
GA	Gallic acid
LAB	lactic acid bacteria
MFS1	Microbial Feed Supplement 1
MFS2	Microbial Feed Supplement 2
OEO	Oregano essential oil
PARL	Particle-associated rumen liquid
PFAs	Phytogenic feed additives
rRNA	Ribosomal Ribonucleic Acid
SARA	Subacute ruminal acidosis
SCFA	short-chain fatty acid
SYRCLE	Systematic Review Centre for Laboratory Animal Experimentation
UDCA	ursodeoxycholic acid
VFAs	Volatile fatty acids
